# Estimation of the true incidence of lactic acidosis within the Lighthouse Clinic cohort, and the likely magnitude of missed diagnoses in the region

**DOI:** 10.7448/IAS.17.4.19558

**Published:** 2014-11-02

**Authors:** Colin Speight, Layout Gabriel, Sam Phiri, Hannock Tweya, Rebecca Sutherland

**Affiliations:** 1 Clinic, Lighthouse Trust, Lilongwe, Malawi; 2 Directorate, Lighthouse Trust, Lilongwe, Malawi; 3 Monitoring and Evaluation, Lighthouse Trust, Lilongwe, Malawi; 4 Edinburgh RIDU, Regional Infectious Diseases Unit, Edinburgh, UK

## Abstract

**Introduction:**

Lactic acidosis is one of the most serious side effects associated with ART, most commonly associated with stavudine. Clinical features are non-specific and specialist laboratory capabilities are essential to confirm the diagnosis, making under-diagnosis likely in resource-constrained settings. Lighthouse Trust is a tertiary referral ART centre with over 23,500 patients on ART. The adjacent University of North Carolina Project laboratory, also serving Kamuzu Central Hospital, has been the only site processing lactate tests in Central Zone for many years. Our objective was to quantify the true incidence within our cohort, and estimate the likely degree of historical missed diagnoses from less central ART clinics.

**Methods:**

All high lactate results between June 2010 and June 2013 were treated as cases, and cross referenced with the Lighthouse database. Patients transferring in to Lighthouse within one month prior to diagnosis were assumed to have been referred due to their lactic acidosis, and moved to the Central Zone cohort to avoid referral bias. Routinely collected quarterly ART cohort data for both Lighthouse and the entire Central Zone were analyzed.

**Results:**

Over the three-year period, from within the Lighthouse cohort, there were 138 cases: 74% were female, median duration on ART was 14 months (IQR 10–26), and 98.5% were attributable to stavudine (only two cases to zidovudine). Over this period, the average number of patients taking stavudine at Lighthouse was 10,960 (3,600 on zidovudine). For the whole Central Zone (minus Lighthouse patients) there were 61,000 on stavudine (4,830 on zidovudine), yet only 124 cases of lactic acidosis were apparently diagnosed from within this cohort.

**Conclusions:**

Although cases may, of course, also have been missed at Lighthouse, as a tertiary referral centre the rate observed is likely to be closer to the true incidence. Over the three years, with 138 cases from the 10,960 patients taking stavudine at Lighthouse, it is likely that somewhere in the region of 700 additional cases occurred amongst the 61,000 patients elsewhere in the Central Zone. This equates to somewhere in the region of 550 missed diagnoses or 80% of all cases. Given that the clinical sequelae of undiagnosed lactic acidosis are either death or at best ART default, this provides further vindication for the decision to phase out stavudine in Malawi.

